# The BulkECexplorer compiles endothelial bulk transcriptomes to predict functional versus leaky transcription

**DOI:** 10.1038/s44161-024-00436-w

**Published:** 2024-03-13

**Authors:** James T. Brash, Guillermo Diez-Pinel, Chiara Colletto, Raphael F.P. Castellan, Alessandro Fantin, Christiana Ruhrberg

**Affiliations:** 1 UCL Institute of Ophthalmology, University College London, London, UK; 2 Department of Biosciences, University of Milan, Milan, Italy

## Abstract

Transcriptomic data can be mined to understand the molecular activity of cell types. Yet, functional genes may remain undetected in RNA sequencing (RNA-seq) experiments for technical reasons, such as insufficient read depth or gene dropout. Conversely, RNA-seq experiments may detect lowly expressed mRNAs thought to be biologically irrelevant products of leaky transcription. To represent a cell type’s functional transcriptome more accurately, we propose compiling many bulk RNA-seq datasets into a compendium and applying established classification models to predict whether detected transcripts are likely products of active or leaky transcription. Here, we present the BulkECexplorer (bulk RNA-seq endothelial cell explorer) compendium of 240 bulk RNA-seq datasets from five vascular endothelial cell subtypes. This resource reports transcript counts for genes of interest and predicts whether detected transcripts are likely the products of active or leaky gene expression. Beyond its usefulness for vascular biology research, this resource provides a blueprint for developing analogous tools for other cell types.

RNA sequencing (RNA-seq) has emerged as a leading method to interrogate the transcriptome of cell populations. Single-cell RNA-seq (scRNA-seq) compendia provide useful resources to distinguish different cell types through their transcriptomic signature and to compare the expression patterns of genes across a range of cell types within organs^1^. By contrast, bulk RNA-seq is widely used to determine average transcript levels in a cell population, for example, to compare transcriptomic changes within a cell type of interest after experimental manipulation^2^ or to correlate transcriptomic and proteomic data^3,4^. More recently, bulk RNA-seq and scRNA-seq data have been combined to map the tumor microenvironment^5^.

A wealth of data on the vascular endothelial cell (EC) transcriptome has been generated using RNA-seq^3,6,7^ and can be mined, for example, through the EndoDB portal^8^, to generate new insights into EC biology. Considering the plethora of proteins that have been implicated in EC signaling pathways, there may be value in examining EC RNA-seq data to confirm that the genes coding for pathway-implicated proteins are indeed expressed in specific endothelial subtypes (for example, ECs of different organs). Although this effort is likely redundant for proteins whose function is defined within a range of EC subtypes, it may be worthwhile for proteins whose roles in ECs are less clear or are controversial. However, some functional genes may not be detected in any individual RNA-seq assay for technical reasons, such as insufficient read depth^9,10^ or gene dropout in scRNA-seq^11,12^. Thus, multiple RNA-seq resources must be examined to gain an accurate overview of the EC transcriptome.

Transcriptomic analysis can be complicated by the presence of low-abundance transcripts that are proposed to be the products of leaky transcription^9,13,14^. Unlike more moderately expressed genes, leakily transcribed genes are not associated with active chromatin markers^15^ and are not thought to be functional within the assayed cell type^9,13,16^. Instead, leaky transcription likely arises when an inactive gene resides near a highly expressed gene that imparts a ‘transcriptional ripple effect’ (refs. 14,17,18). Several computational methods have been proposed for identifying leaky transcripts in bulk RNA-seq data^9,13,19,20^. However, to the best of our knowledge, no study has systematically applied these methods to a collection of bulk RNA-seq datasets for one cell type, nor has it been examined whether this approach could be used to systematically distinguish actively transcribed from leakily transcribed genes in ECs.

Here, we have compiled a compendium of 240 publicly available bulk RNA-seq datasets from five human and mouse EC subtypes that are commonly used for vascular biology research. Beyond providing a convenient resource for summarizing transcript counts from datasets that meet strict inclusion criteria for data quality and EC identity, we have applied previously validated classification models to these datasets to predict whether detected transcripts are likely the products of active or leaky transcription. This freely available resource is termed the BulkECexplorer (bulk RNA-seq EC explorer) and can be explored without prior bioinformatics expertise. We have illustrated the utility of the BulkECexplorer by interrogating a gene family with known vascular functions and evaluated the predictive value of our tool with a systematic confusion matrix-based approach. We propose the BulkECexplorer as a convenient and reliable resource for evaluating whether genes of interest are actively expressed in primary human or mouse ECs to help prioritize genes for further functional investigation.

## Results

### BulkECexplorer construction

**Fig. 1:**
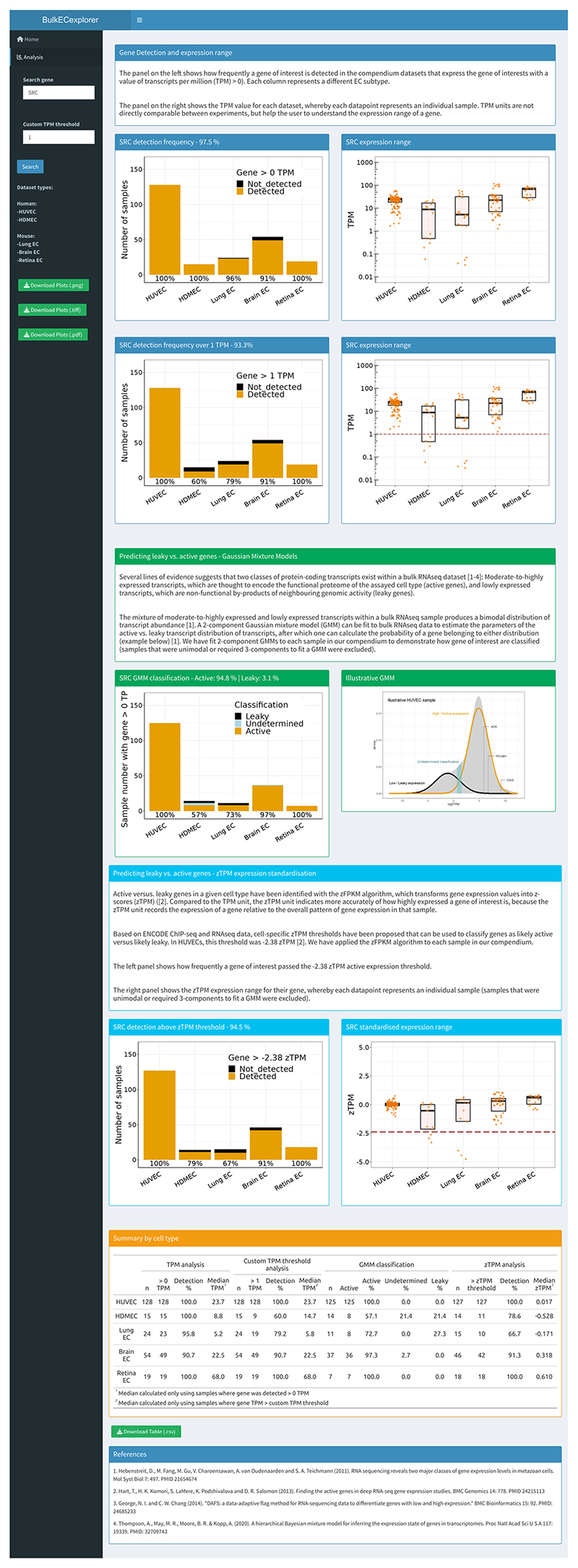
BulkECexplorer online app display. The image shows a snapshot of the output of the BulkECexplorer when queried for a gene of interest (for example, *SRC*). The blue section displays the gene detection rate and expression range. Top left box, stacked bar chart depicting the number of datasets with *SRC* >0 TPM, resolved by EC subtype. The percentage of datasets with *SRC* >0 TPM in each EC subtype is reported below each bar. The percentage of datasets with *SRC* >0 TPM across all datasets, independently of subtype, is reported above the bar graph. Top right box, boxplots of *SRC* TPM values for individual datasets, resolved by EC subtype, including the median (center line). The bottom boxes show the corresponding data with a default ‘>1 TPM’ expression threshold that can be customized. The red dashed line (bottom right box) indicates the 1 TPM gene expression threshold. The green section summarizes data obtained by predicting leaky versus active genes using GMMs. Left box, stacked bar chart depicting the number of datasets in which *SRC* expression was classified as active, leaky or undetermined, resolved by EC subtype. The percentage of datasets in which *SRC* expression was classified as active in each EC subtype is reported below each bar. The percentage of datasets in which *SRC* expression was classified as active versus leaky across all datasets is reported above the bar chart. Right box, GMM for a representative HUVEC dataset; expression values for three core EC genes are indicated. The cyan section summarizes data obtained by predicting leaky versus active genes using zTPM expression standardization for each dataset. Left box, stacked bar chart depicting the number of datasets in which *SRC* expression was above the −2.38 zTPM threshold, resolved by EC subtype. The percentage of datasets in which *SRC* was detected above the threshold in each EC subtype is reported below each bar. The percentage of datasets in which *SRC* was detected above the threshold across all datasets is reported above the bar chart. Right box, boxplots of *SRC* zTPM values for individual datasets, resolved by EC subtype, including the median (center line). The red dashed line indicates the −2.38 zTPM gene expression threshold. The orange section provides a summary by cell type for the number of datasets analyzed per EC subtype alongside outputs for the analysis of TPM values and the GMM versus zTPM predictions.

Bulk RNA-seq provides an average measure of gene expression in a cell population, but some expressed genes may not be detected in any single experiment for technical reasons. For this reason, we compiled publicly available endothelial bulk RNA-seq data into a compendium for a more accurate overview of gene expression. Thus, we queried the European Nucleotide Archive (ENA) for bulk RNA-seq data of five EC subtypes commonly used for vascular biology research: human umbilical vein ECs (HUVECs), human dermal microvascular ECs (HDMECs), primary mouse lung ECs, primary mouse brain ECs and primary mouse retina ECs. Our search returned 195 sequencing projects, each containing multiple RNA-seq runs. After imposing strict exclusion criteria (Methods), a total of 264 RNA-seq runs with unique sample identifiers were downloaded and aligned to the human or mouse genome, as appropriate (Supplementary Table 1). After quantifying transcript abundance, 24 datasets were excluded from analysis because they lacked (or had low levels of) transcripts for the core endothelial markers *KDR* and *CDH5*, comprised low read numbers, or had poor read alignment. Thus, we selected a total of 240 endothelial bulk RNA-seq datasets from 59 sequencing projects (Supplementary Table 1) and compiled them into a compendium that is searchable through an online interface termed the BulkECexplorer (https://ruhrberglab.shinyapps.io/BulkECexplorer/). For each queried gene, the BulkEC-explorer reports the number and proportion of datasets containing transcripts and their expression range, both per EC subtype and across all datasets (Fig. 1, blue section). The BulkECexplorer also enables the presentation of the corresponding values with a manually adjustable TPM (transcripts per million) threshold (Fig. 1, blue section).

As bulk RNA-seq can detect lowly expressed transcripts that may be the nonfunctional products of leaky transcription, Gaussian mixture models (GMMs) and the zFPKM algorithm have been used to predict leaky versus active transcripts in bulk RNA-seq data from several different non-EC types^9,13,14^. Here, we have applied these classification models to all eligible datasets in the BulkECexplorer (Methods). The GMM- and TPM-based predictive data of leaky versus active transcription are reported for each queried gene individually per EC subtype and across all datasets analyzed (Fig. 1, green and cyan sections, respectively). A summary table displays the uninterpreted expression data in TPM alongside predictive data per EC subtype (Fig. 1, orange section). All data are downloadable in a graphic (.png,.tiff,.pdf) or tabular format.

To illustrate the utility of the BulkECexplorer, we examined the expression of SRC family kinases (SFKs), which were selected as a use case because some family members have experimentally proven endothelial roles, whereas others are deemed specific to hematopoietic cell types. For example, signaling through the SFKs SRC, YES1, FYN and LYN has been described in all EC subtypes included in the BulkECexplorer, including HUVECs^21,22^, HDMECs^23^, mouse lung ECs^21,22,24^, mouse brain ECs^25^ and mouse retina ECs^26,27^. By contrast, the SFKs FGR, LCK, HCK and BLK are expressed and functional in hematopoietic cells^28–32^. Thus, we compared the detection of endothelial and hematopoietic SFKs in scRNA-seq datasets of multiple organs to BulkECexplorer results. Next, we further investigated whether the BulkECexplorer accurately predicts which SFKs are functional in ECs.

### SFK detection in endothelial scRNA-seq datasets

We first examined scRNA-seq-based SFK detection in brain, heart and lung ECs using the EC atlas^33^ and Tabula Muris^34^ before corroborating findings with the Mouse Vascular Single Cells database^6^. We also extended our analysis to scRNA-seq datasets from the human dermis^35^ and trachea^36^. In all these organs, YES1, SRC, FYN and LYN have functions in ECs, including the regulation of angiogenesis or vascular permeability^23,25,37^.

**Fig. 2:**
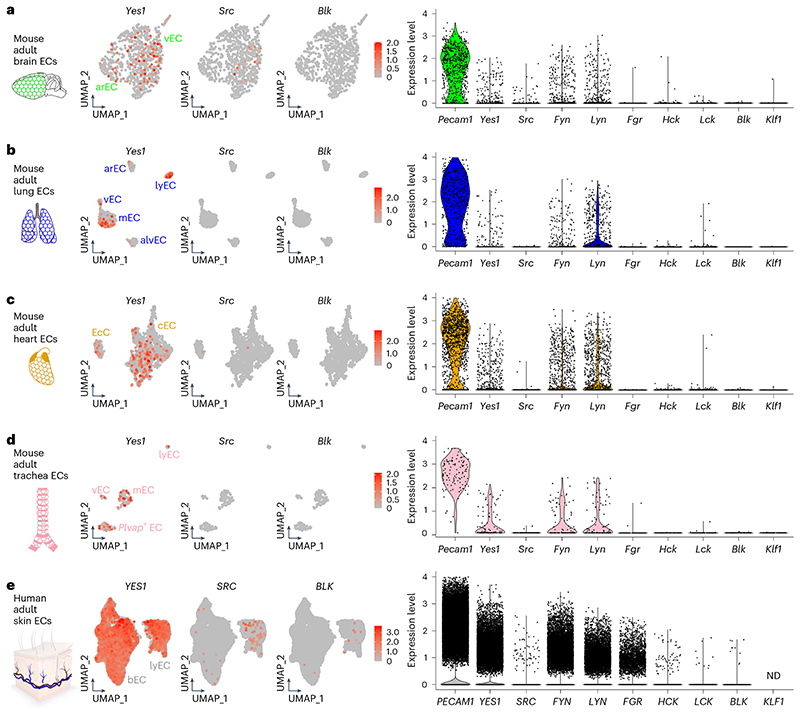
SFK transcript detection in EC scRNA-seq data from mouse and human organs. **a−d**, Analysis of Tabula Muris scRNA-seq data from mouse adult brain (**a**), lung (**b**), heart (**c**) and trachea (**d**). **e**, Analysis of scRNA-seq data of FACS-captured PECAM1-positive cells from the human adult dermis. After the selection of the EC subsets, UMAP and violin plots were generated to compare *Yes1, Src, Fyn, Lyn, Fgr, Hck, Lck* and *Blk* transcript levels; the violin plots also show *Pecam1* and *Klf1* transcript levels as positive and negative EC markers (for raw data, see the corresponding source data file). Each data point represents the value for one cell. ND, not detected. arEC, arterial EC; alvEC, alveolar EC; bEC, blood EC; cEC, cardiac EC; EcC, endocardial cell; lyEC, lymphatic EC; mEC, microvascular EC; vEC, venous EC.

The EC atlas was obtained by sequencing fluorescence-activated cell sorting (FACS)-isolated platelet and EC adhesion molecule 1 (PECAM1)-positive ECs^33^. *Yes1, Fyn* and *Lyn* were detected in 12−30% of brain, lung and heart EC populations, whereas transcripts for *Fgr, Hck, Lck* and *Blk* were not identified in these populations (Tables 1 and 2). Unexpectedly, ECs with *Src* transcripts were not identified in the heart or lung; they were rare in the brain, where they also had low *Src* transcript levels (Tables 1 and 2). In Tabula Muris, whole-organ single-cell suspensions were analyzed by FACS/Smart-seq2 and a droplet-based approach with 10x Genomics; here, we analyzed the FACS/Smart-seq2-based dataset because it has superior transcriptome coverage per cell compared to the droplet dataset^34^. Tabula Muris also contains trachea data, which are not included in the EC atlas. For each organ, ECs were identified as described^38^. *Yes1, Fyn* and *Lyn* were detected in 17−45% of mouse brain, lung and heart ECs and in 41−50% of mouse trachea ECs (Tables 1 and 2 and Fig. 2a-c). In most organs, *Fgr* and *Blk* were detected in only approximately 1%, and *Hck* and *Lck* were detected in 3−5% of ECs (Tables 1 and 2 and Fig. 2a−d). The proportion of ECs with detectable *Src* was similarly low (*Src* detection rate: 5.6% in brain ECs, 1.2% in lung ECs, 0.5% in heart ECs, 1.8% in trachea ECs; Table 1 and Fig. 2a-d). In the lung, heart and trachea, this proportion was almost as low as that of ECs expressing *Klf1*, an erythroid marker used as an example for a gene not expected to be transcribed in ECs (Table 1 and Fig. 2b−d). We then interrogated lung and brain scRNA-seq data from the Mouse Vascular Single Cells database through its online interface^6^ (data discoverable at http://betsholtzlab.org/VascularSingleCells/database.html). We again observed a higher proportion of ECs with *Src* transcripts and higher average levels of *Src* transcripts in the brain than in the lung; however, the number of ECs with detectable *Src* transcripts and the average transcript counts were very low compared to data for *Yes1, Fyn* or *Lyn*.

Human adult dermal EC scRNA-seq data were obtained from FACS-isolated PECAM1-positive dermal ECs, which form a larger cluster of blood vascular ECs and a smaller cluster of lymphatic vascular ECs^35^. We readily detected *YES1* in ECs of both clusters, but *SRC* transcripts were rarely detected and mostly resided in the lymphatic rather than blood vascular EC cluster (Tables 1 and 2 and Fig. 2e). Transcripts for *FYN, LYN* and *FGR* were readily detected in dermal ECs, similar to *YES1*; in contrast, *HCK, LCK* and *BLK* were poorly detected, similar to *SRC* (Fig. 2e and Tables 1 and 2). In the human trachea dataset^36^, we selected the EC cluster according to its expression of core endothelial genes, including *PECAM1* (Tables 1 and 2). *YES1* was detected in most ECs, but *SRC, FYN* and *LYN* were detected in much fewer ECs (detection rates: *YES1* 75.2%, *SRC* 11.3%, *FYN* 5.5% and *LYN* 3.6%; Table 1). Although *SRC* was detected in more ECs than other SFK genes, except *YES1* (Table 1), the average *SRC* transcript levels were lower than those for *YES1, FYN, LYN* and *FGR* and more similar to those for *HCK, LCK* and *BLK* (Table 2).

Taken together, mining of mouse and human scRNA-seq EC datasets showed that *YES1, LYN* and *FYN* were widely expressed in ECs, whereas *SRC* transcripts were detected at low levels in only a few brain ECs and at even lower levels and more rarely in ECs of other organs. Notably, *SRC* transcripts were lacking from the EC atlas, except in the brain dataset. These findings were surprising due to SRC’s widely accepted endothelial functions^25,39,40^. Therefore, we interrogated the BulkECexplorer for endothelial expression of SFKs, including SRC.

### SFK detection in endothelial bulk RNA-seq datasets

**Fig. 3:**
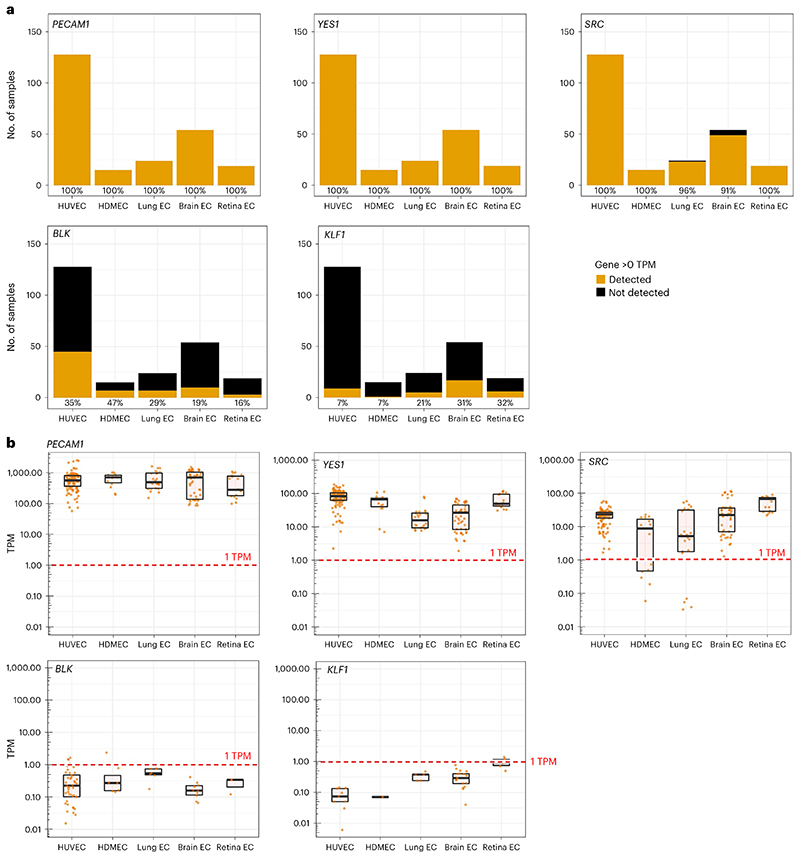
SFK transcript detection in bulk RNA-seq data from primary human and mouse ECs. Number of datasets analyzed across EC subtypes for each of the five indicated genes, *n* = 240. **a**, Stacked bar charts depicting the total number of datasets and the frequency at which transcripts for the indicated genes were detected (>0 TPM) or not detected (0 TPM), resolved by EC subtype (HUVEC *n* = 128, HDMEC *n* = 15, mouse lung EC *n* = 24, mouse brain EC *n* = 54, mouse retina EC *n* = 19). **b**, Transcript levels for the indicated genes with expression >0 TPM in each dataset for the indicated EC subtypes, including boxplots to illustrate the median (center line) and interquartile range (box limits) (for *n*, see the corresponding source data file); each data point represents one dataset. The red dashed line indicates the 1 TPM threshold, a commonly used albeit heuristic transcript level above which a gene is considered to be expressed at a level that may be biologically relevant.

Consistent with scRNA-seq analysis, the BulkECexplorer robustly detected *FYN, LYN* and *YES1* in all five EC subtypes, with *FYN* and *YES1* detected in all 240 datasets and *LYN* detected in 239 of the 240 datasets (Fig. 3a and see data in the BulkECexplorer). Despite a low detection rate in scRNA-seq analyses, *SRC* transcripts were detected in the vast majority (234/240) of BulkECexplorer datasets, albeit with varying expression levels (Fig. 3a,b; data resolved by EC subtype). In mouse brain and retina EC datasets, *Src* transcript levels were similar to those of *Yes1*; in contrast, *SRC* transcript levels were lower than those of *YES1* in human ECs and most mouse lung EC datasets (Fig. 3b). In 5 of the 24 lung EC datasets, 5 of the 54 brain EC datasets and 6 of the 15 HDMEC datasets, SRC was expressed at <1 TPM, which is a commonly used, albeit heuristic, threshold when selecting genes for downstream analysis. The six datasets lacking *SRC* transcripts were derived from primary mouse brain and lung ECs (Fig. 3a,b).

We next used the BulkECexplorer to examine the expression of hematopoietic SFKs with no known function in ECs. *FGR, HCK* and *LCK* were detected in 53.8%, 32.5% and 39.2% of ECs, respectively, although at <1 TPM for most datasets (*FGR* 210/240, *HCK* 192/240, *LCK* 200/240). Nevertheless, some datasets contained a higher number of transcripts for these SFKs, including *FGR* in HDMEC datasets, *HCK* in retina datasets and *LCK* in lung datasets (data discoverable in the BulkECexplorer). The functional relevance of this EC subtype-specific transcript enrichment is unknown. Unexpectedly, the B cell-specific SFK *BLK*^28^ was detected in 30% of BulkECexplorer datasets, but at low levels (<1 TPM), except in five human datasets with a transcript level of 1−2.5 TPM (Fig. 3b). This distribution was somewhat similar to that of *KLF1*, which was detected in 15.8% of BulkECexplorer datasets, mostly at low levels (<1 TPM) (Fig. 3b). Low expression of genes in cells in which they are not expected to function has previously been attributed to ‘leaky’ transcription, possibly driven by the expression of nearby highly expressed genes^9,13,14^.

In summary, our SFK analysis corroborates that the BulkEC-explorer allows comparing gene expression between EC subtypes, including reporting the expression characteristics of transcripts that have a low detection rate with scRNA-seq. On the one hand, our analysis confirmed that SRC is robustly expressed in ECs despite poor detection by scRNA-seq analysis. On the other hand, the detection of >0 TPM as a measure of gene expression could not reliably predict whether low transcript levels in ECs, such as those for hematopoietic genes, reflect leaky transcription of nonfunctional genes.

### SFK transcript classification with the BulkECexplorer

**Fig. 4:**
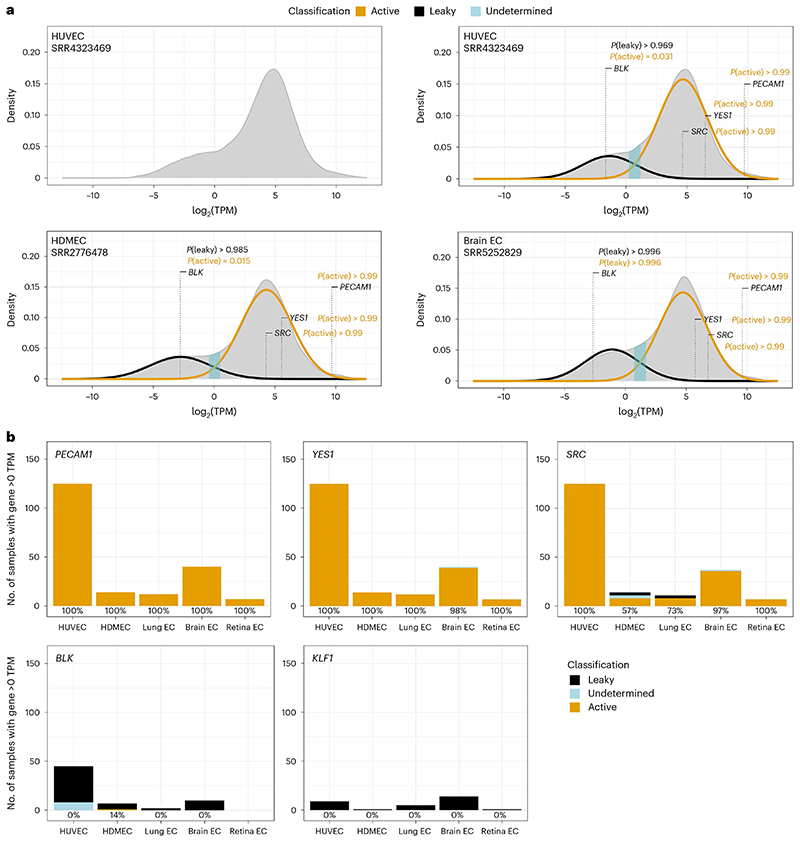
GMM-based classification predicts that *SRC* but not *BLK* is actively expressed in ECs. **a**, Illustrative kernal density estimates (KDEs) of log_2_-transformed TPM values for protein-coding genes in the bulk RNA-seq data from the indicated datasets. Expectation maximization was used to estimate the parameters of the low and high Gaussian distributions (predicted leaky versus active transcription), represented by black and gold fit curves, respectively. The log_2_(TPM) and *P*(active) values for *PECAM1, YES1, SRC* and *BLK* in each dataset are indicated together with the *P*(leaky) values for *BLK*. The illustrative HUVEC dataset is also shown without the estimated Gaussian distributions to its transcript distribution (top left). **b**, Stacked bar charts depicting the number of datasets per EC subtype in which the indicated genes were classified by the GMM method as active, leaky or undetermined, resolved by EC subtype; lung, brain and retina EC data were obtained from mouse datasets. The percentage of datasets in which each gene was classified as actively expressed is reported for each EC subtype below each bar (number of eligible, bimodally distributed datasets: *PECAM1 n* = 198, *YES1 n* = 198, *SRC n* = 194, *BLK n* = 64, *KLF1 n* = 30). Note that only datasets with a transcript level of >0 TPM are classified; therefore, datasets not shown have a transcript level of <0 TPM.

To predict whether transcripts are products of active or leaky transcription, we applied GMM classification^13^ and the zTPM^9^ algorithm to the BulkECexplorer datasets. The GMM classification approach is based on prior work showing that the mixture of protein-coding transcripts from leakily and actively transcribed genes produces a bimodal distribution of transcript abundance in a homogeneous population of mammalian cells^13^. Actively expressed genes form a dominant Gaussian distribution in the higher expression range, whereas leakily expressed genes form a less prominent Gaussian distribution in the lower expression range^13^. The overlap between the two distributions produces a dominant right peak with a characteristic ‘left shoulder’ instead of two readily discernible distributions^13^. We established that such bimodal distributions were observable in EC bulk RNA-seq data (Fig. 4a, top left). As done for other cell types^9,13,14^, the parameters of the two transcript distributions can be estimated by fitting a two-component GMM to the expression data of each BulkECexplorer dataset individually.

We could fit a two-component GMM to 98% of HDMEC and HUVEC datasets and 61% of mouse EC datasets (examples in Fig. 4a; total: 198/240 datasets). A further 23% of mouse datasets presented a bimodal distribution with some degree of a left shoulder, but a third component was required to fit a GMM; these datasets were excluded from further analysis because the nature of a third Gaussian distribution is undefined within the context of leaky versus active transcription. Other mouse EC datasets appeared unimodal, without evidence of a left shoulder; as a unimodal distribution can be due to transcripts from a contaminating cell type^13,41^, these datasets may reflect the technical pitfalls of separating a relatively small EC population from other dominant cell types in small mouse organs. Thus, we restricted our analysis to the 198 human and mouse datasets (hereafter referred to as ‘eligible’ datasets) with a bimodal transcript distribution to which we could fit a two-component GMM, indicative of leaky and active expression distributions. In each dataset, we classified genes as actively expressed if the probability of belonging to the high distribution, termed *P*(active), was >0.67, as leakily expressed if *P*(active) was <0.33, and as undetermined if *P*(active) was between these thresholds. The ‘undetermined’ classification was applied to genes for which the classification was probabilistically less definitive, that is, *P*(active) < 0.67 and *P*(leaky)>0.33.

Similar to the established EC marker *PECAM1, FYN* and *LYN* were classified as actively expressed in 198 of 198 datasets, whereas *YES1* and *SRC* were classified as actively expressed in most eligible datasets in which they were expressed (197/198 and 184/194, respectively) (Fig. 4b; data resolved per EC type). Thus, BulkECexplorer GMM analysis correctly classified SFKs with known EC functionality as actively expressed, including SRC. We next examined hematopoietic SFKs for which the BulkECexplorer had detected low-level EC expression in some datasets and found that BulkECexplorer GMM analysis largely classified these genes, when they were detected, as not actively expressed in ECs. Thus, *FGR* was classified as actively expressed in 6.9% of 102, *HCK* in 28.3% of 53 and *LCK* in 16.7% of 66 datasets in which they were detected (data discoverable in the BulkECexplorer). Transcripts from the B cell gene *BLK*^28^ were classified as active in only 1.6% of 64 datasets in which they were detected, similar to *KLF1* (Fig. 4b; data resolved per EC type). Other examples of non-EC genes that were detected in some bulk RNA-seq EC datasets but then largely classified as not actively expressed include another erythroid gene (*RHD*), ocular genes (*LENEP, CRYBB2*), osteoblast genes (*BGLAP, DMP1*) and several sex cell-specific genes (*DDX4, GDF9, YBX2, SPACA4*) (data discoverable in the BulkECexplorer). These findings support the validity of the GMM approach for classifying active versus leaky gene expression in EC bulk RNA-seq data.

**Extended Data Fig. 1|:**
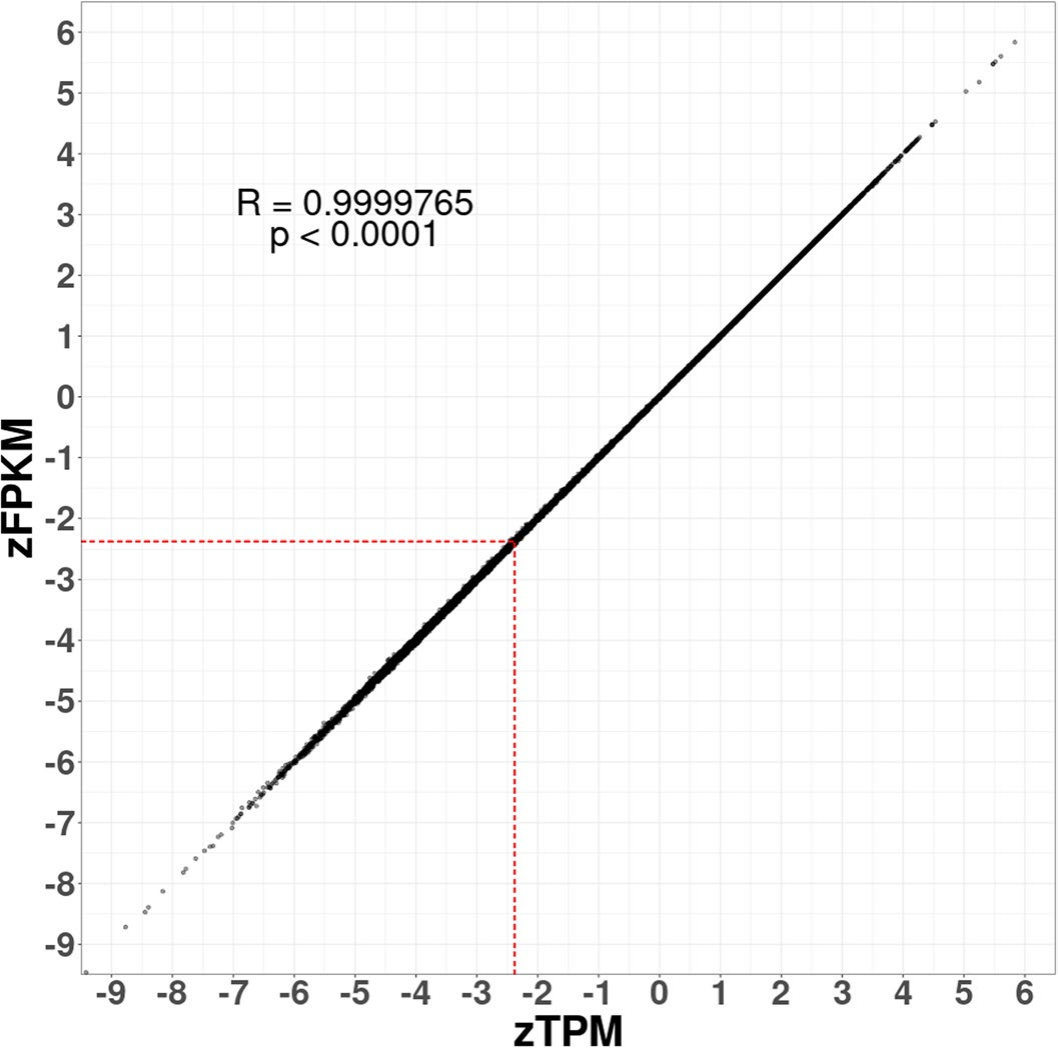
zTPM correlates with zFPKM. Correlation of zTPM and zFPKM units for a selection of 146 vascular, neuronal, glial, immune and bone genes from 220 bulk RNA-seq datasets (unimodal datasets were not analysed). R, Pearson correlation coefficient, two sided p value0 TPM were plotted (n = 18784). The red stippled line indicates the published threshold for leaky gene expression of −2.38 zFKPM for HUVEC is transposed into a corresponding TPM threshold.

**Fig. 5:**
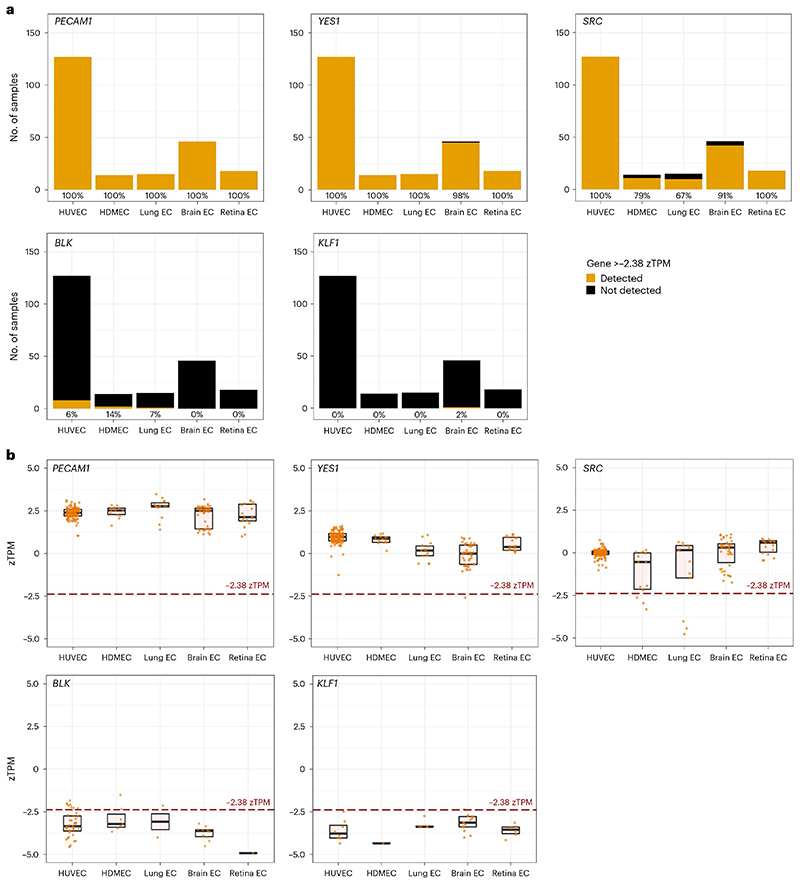
zTPM standardization predicts that *SRC* but not *BLK* is actively expressed in ECs. Number of datasets analyzed across EC subtypes for each of the five genes, *n* = 220. **a**, Stacked bar charts depicting the total number of datasets per EC subtype in which each indicated gene was detected (>−2.38 zTPM) or not detected (≤−2.38 zTPM) above the previously determined active expression threshold of −2.38 zTPM for HUVECs. The percentage of datasets in which the indicated gene was expressed above the threshold is reported below each bar for the corresponding EC subtype. HUVEC *n* = 127, HDMEC *n* = 14, mouse lung EC *n* = 15, mouse brain EC *n* = 46, mouse retina EC *n* = 18. **b**, zTPM values for the indicated genes in each dataset with expression >0 TPM, split by EC subtype. The TPM values for each dataset are available in the corresponding source data file. Each data point represents one dataset; values are shown together with boxplots to illustrate the median (center line) and interquartile range (box limits) (for *n*, see the corresponding source data file). The red dashed line indicates the −2.38 zTPM threshold above which a gene is considered actively expressed in HUVECs. Lung, brain and retina EC data are from mouse datasets.

The zFPKM algorithm provides an alternative method for predicting whether transcripts in bulk RNA-seq data are products of active or leaky transcription^9^. With this algorithm, gene expression values (in fragments per kilobase million (FPKM)) are transformed into *z* scores (zFPKM) based on the parameters of an active expression Gaussian distribution fitted around the peak of the gene expression distribution for protein-coding genes^9^ (Methods). Thus, zFPKM provides a standardized measure of gene expression relative to the global pattern of gene expression in a dataset^9^. In the original study describing the zFPKM algorithm^9^, epigenomic and RNA-seq data from the ENCODE project were used to calculate a selection of cell-specific zFPKM thresholds at which genes are more frequently associated with active rather than repressive chromatin markers indicative of actively transcribed versus leakily transcribed genes, respectively^42,43^. For HUVECs, the only EC type for which the zFPKM threshold has been determined, the threshold was −2.38 zFPKM (ref. 9). The strong correlation between zTPM and zFPKM values (Extended Data Fig. 1) allowed us to adopt the −2.38 threshold after the zTPM transformation of each BulkEC-explorer dataset (Methods). *PECAM1* and *FYN* exceeded the −2.38 zTPM threshold in all eligible datasets (220/220), *YES1* and *LYN* in 99.5% (219/220) and *SRC* in 94.5% (208/220) (Fig. 5a,b and data discoverable in the BulkECexplorer). By contrast, *FGR, HCK, LCK* and *BLK* exceeded this threshold in only 6.4%, 19.1%, 12.3% and 5%, respectively, and *KLF1* transcripts in only 0.5% of the datasets in which their transcripts were detected (Fig. 5a,b and data discoverable in the BulkECexplorer).

In summary, transcripts for hematopoietic SFKs were either not detected by the BulkECexplorer or, when detected, predominantly classified as the products of leaky transcription in ECs. Instead, *SRC* was classified as actively expressed, similar to *YES1, FYN* and *LYN*, agreeing with known EC functions. Therefore, the use case of the SFKs reinforces that the BulkECexplorer helps predict whether EC transcripts poorly detected by scRNA-seq or detected at low levels by bulk RNA-seq are likely functional in ECs.

### Systematic evaluation of BulkECexplorer transcript classification

Next, we compared the predictive value of the BulkECexplorer’s GMM- and zTPM-based classifications to classifications based on transcript levels alone (‘transcript level >0 TPM’ and the commonly used but heuristic threshold ‘transcript level >1 TPM’). For this, we built a confusion matrix with widely used markers for ECs (‘actual positives’, *n* = 37) versus non-EC populations, including immune cells, neurons, glial cells and bone cells (‘actual negatives’, *n* = 109; Supplementary Table 2). We scored the BulkECexplorer predictions of actively expressed (‘predicted positives’) and leakily expressed (‘predicted negatives’) genes against these 146 markers in all gene−dataset combinations across all eligible datasets for each classification approach (Supplementary Table 3). Genes with TPM = 0 were scored as predicted negatives, whereas gene−dataset combinations classified by GMM as ‘undetermined’ were excluded (but are included in the BulkECexplorer online results). As the four classification methods assessed here each draw on a varying number of eligible datasets (see above), the reported performance results are valid only in the context of the BulkECexplorer.

**Extended Data Fig. 2|:**
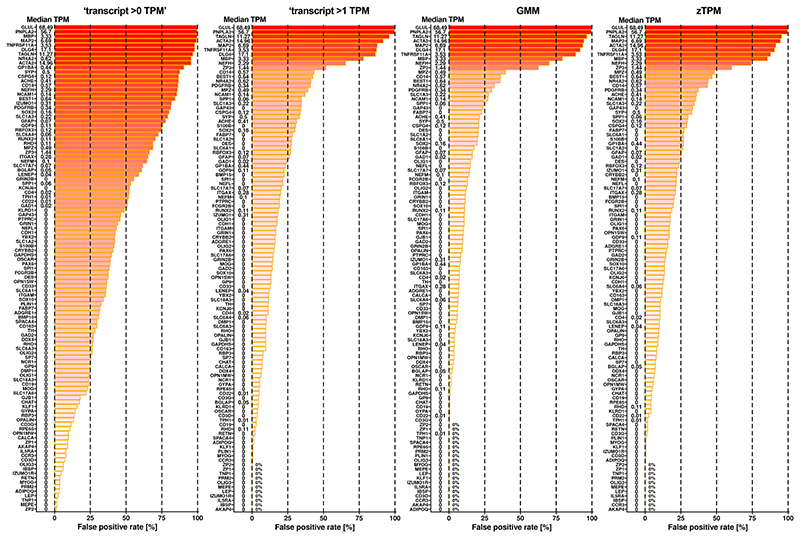
False positive detection by the four BulkECexplorer classification methods. The false positive rate for each of the 1st run *actual negative* markers (n = 109) is shown for each of the classification methods and across all eligible datasets in the BulkECexplorer (transcript level >0 TPM, transcript level >1 TPM, GMM, zTPM). Gene names are shown adjacent to the Y axis alongside their median TPM.

**Extended Data Fig. 3|:**
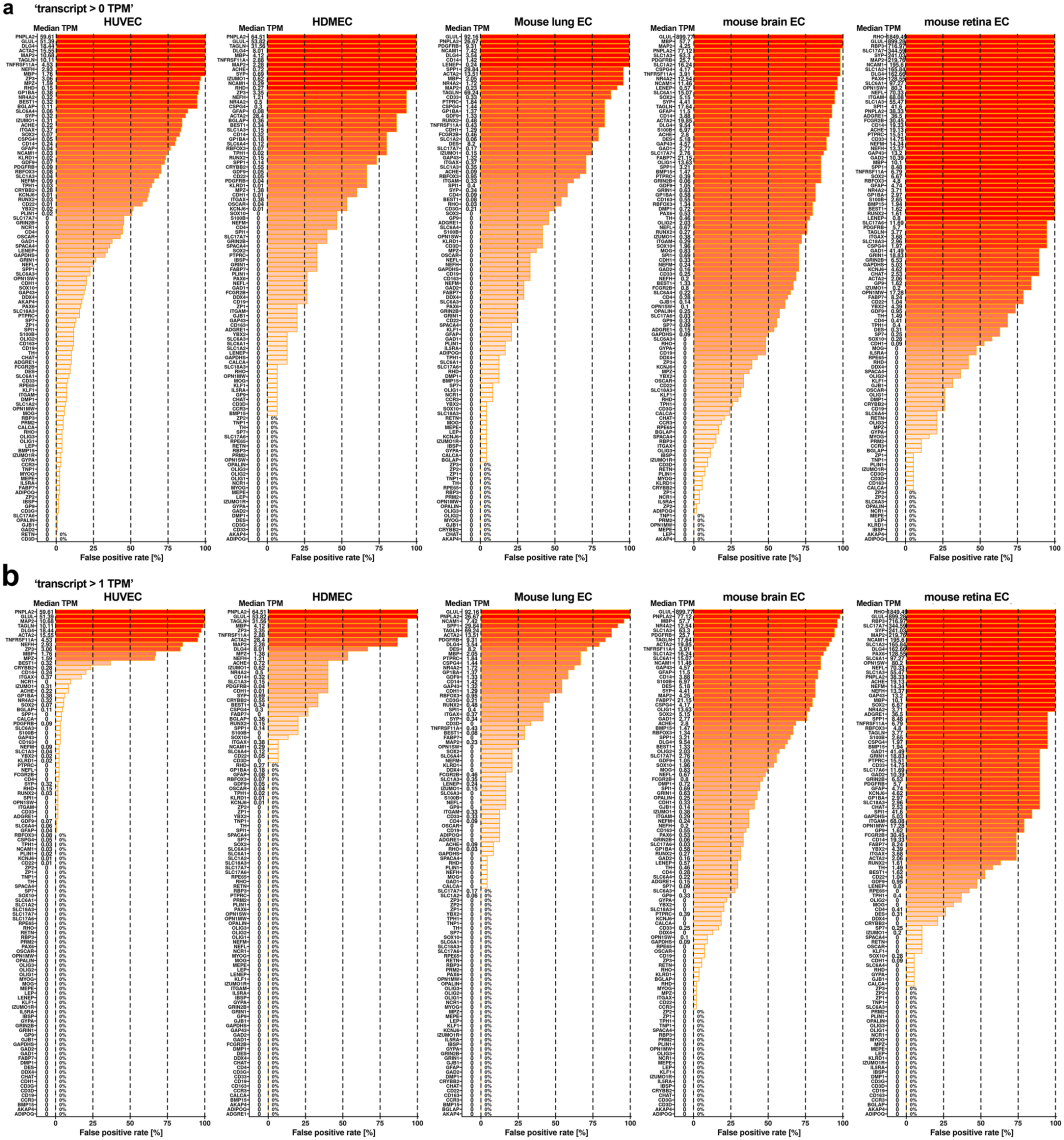
False positive detection based on transcript abundance alone, resolved by EC subtype. The false positive rate for each of the 1st run *actual negative* markers (n = 109) for each of the five EC subtypes and across all eligible datasets in the BulkECexplorer, using the ‘transcript level >0 TPM’ (**a**) and ‘transcript level >1 TPM’ (**b**) approaches. Gene names are shown adjacent to the Y axis alongside their median TPM.

**Extended Data Fig. 4|:**
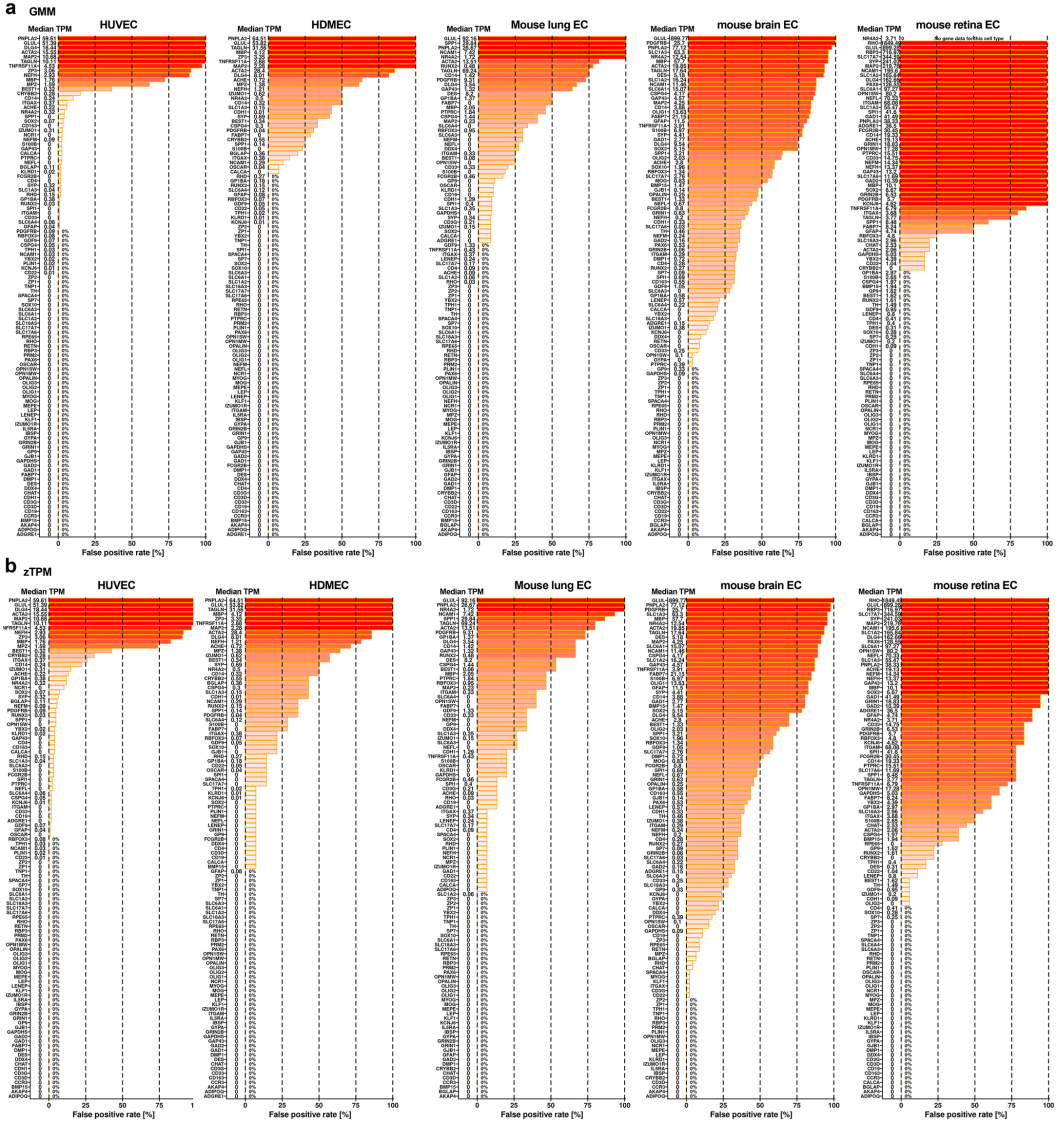
False positive detection with GMM and zTPM approaches, resolved by EC subtype. The false positive rate for each of the 1st run *actual negative* markers (n = 109) for the five EC subtypes and across all eligible datasets in the BulkECexplorer using the GMM **(a)** and zTPM **(b)** approaches. Gene names are shown adjacent to the Y axis alongside their median TPM.

In our primary analysis, all four classification approaches had similarly high ‘sensitivity’ scores (true positive rate; Table 3). Using detection alone for classification (transcript >0 TPM) had the lowest ‘specificity’ scores (true negative rate; Table 3), presumably because this classification returns many genes with very low transcript levels that belong to the leaky EC transcriptome. Indeed, we detected transcripts for 19,436 genes out of a possible total of 19,878 protein-coding genes in the BulkECexplorer’s HUVEC datasets. Compared to ‘transcript >0 TPM’, the GMM, zTPM and ‘transcript >1 TPM’ approaches had higher specificity scores (true negative rate; Table 3), with reduced false-positive rates across all EC subtypes and for each EC subtype individually (Extended Data Figs. 2−4). Although the GMM classification performed slightly better than the zTPM and ‘transcript >1 TPM’ classifications (Table 3), the confusion matrix scores suggest that the GMM, zTPM and ‘transcript >1 TPM’ approaches can all be used to predict leakily expressed genes without substantial losses in identifying actively expressed genes.

A conceptual limitation of accurately selecting markers for the actual negatives list is the uncertainty of whether a marker for another cell type is indeed nonfunctional in ECs. For example, we a priori excluded the neural marker nestin (*Nes*) because we know that it is present in embryonic and neovascular ECs in vivo^44,45^. As the BulkEC-explorer consistently detected robust transcript levels for *Nes* and several other non-EC markers in our list of actual negatives (Extended Data Table 1 and Extended Data Figs. 2−4), we cross-referenced our actual negatives list against a published HUVEC proteome^46^. Transcripts from six genes in our list were present in the HUVEC proteome, namely the adipose marker *PNPLA2*, the smooth muscle marker *TAGLN*, and the neuroglial markers *MAP2, GAD1, GLUL* and *GAPDHS* (Extended Data Table 1). BulkECexplorer analysis corroborated that *PNPLA2, TAGLN, MAP2* and *GLUL* transcripts were expressed at levels >10 TPM in HUVECs and also classified these genes as actively expressed in one or more of the other EC subtypes (Extended Data Table 1). After removing these HUVEC proteome-expressed markers from the actual negatives list for a refined assessment, specificity was increased across all classification approaches for all EC subtypes (Table 3).

Overall, specificity scores (Table 3) were higher for HUVECs, HDMECs and mouse lung ECs than for ECs from the mouse brain or retina, where ECs interact with neurons, glia and pericytes in the neurovascular unit^47^ and also with microglia^48^. Notably, several known markers for these EC-interacting cell types were present in brain and retina EC datasets at levels predicted to reflect active transcription, such as the microglia marker *ITGAM*, the astrocyte marker *GFAP*, the oligodendrocyte marker *OPALIN* and the neural cell marker *SOX2*; in contrast, they were not detected or were predicted to be leakily expressed in cultured human ECs (Extended Data Table 1). Moreover, pericyte and/or vascular smooth muscle cell markers, such as *DES*, were detected in mouse brain, retina and lung ECs at levels predicted to reflect active transcription (Extended Data Table 1). The presence of transcripts from EC-interacting cell types may reflect EC dataset contamination with parenchymal cells or may corroborate the idea that ECs endogenously contain transcripts typical of neighboring cell types^7^. To model the predictive functionality of the tool with these factors isolated, we removed markers for EC-interacting cell types from the list of ‘negative’ genes for a third run and observed increased specificity scores for all EC subtypes, with scores for mouse brain and retina ECs now more similar to those for lung and cultured ECs (Table 3). These findings advocate further investigation to understand why transcripts of EC-interacting cell types can be abundant in EC bulk RNA-seq data.

## Discussion

Here, we show that the BulkECexplorer provides an effective tool to interrogate gene expression data across five EC subtypes commonly used for functional studies in vascular biology research. Supporting its reliability, the BulkECexplorer consistently detected transcripts for SFKs with known EC functions (*YES1, SRC, FYN* and *LYN*), although *SRC* was expressed at lower levels than *YES1, LYN* or *FYN* in some EC subtypes, consistent with prior microarray analysis^49^. For *SRC*, BulkECexplorer analysis returned different results to scRNA-seq datasets, which lacked counts for *SRC* transcripts or detected them either at very low levels or infrequently in ECs from most organs, despite SRC’s well-established EC functions. Thus, we suggest that the BulkECexplorer can complement scRNA-seq-based analysis of EC gene expression while also providing insight into gene expression in EC subtypes not typically analyzed by scRNA-seq but commonly used for in vitro research (for example, HDMECs, HUVECs).

An interesting observation was the low-level expression of non-EC SFKs within many datasets of the BulkECexplorer. Furthermore, the detection of protein-coding transcripts for 19,436 genes across 128 HUVEC datasets in the BulkECexplorer was reminiscent of the prior finding that >20,000 protein-coding or processed transcripts were detected in B cell bulk RNA-seq data^10^. These observations agree with the idea that most genes can be transcribed in a given cell type^50^. Such expansive gene expression may appear difficult to reconcile with the concept of a cell-specific transcriptome unless it is considered that many unexpected transcripts are detected at very low levels. Thus, protein-coding transcripts in a homogeneous cell population can be assigned to a higher-expressed (HE) class encoding the functional proteome of that cell type and a lower-expressed (LE) class that is proposed to be nonfunctional and caused by leaky transcription, akin to biological noise^9,13^. A two-class model for gene expression is supported by epigenomic and proteomic evidence, which shows that LE genes, unlike HE genes, lack epigenetic markers of active transcription^9,51^ and that their protein products are poorly detected by mass spectrometry^16,52,53^. Notably, a two-class gene expression distribution, as shown here for ECs, has previously been reported for a range of other cell types and in multiple species, including normal fibroblasts, epithelial cells, immune cells, neurons and transformed cell lines^9,13,15,16,51–55^.

Applying GMM- or zTPM-based approaches to BulkECexplorer datasets to predict whether genes belong to the HE (active) or LE (leaky) distributions classified *SRC* transcription as active and not leaky, although some heterogeneity was observed for HDMECs and lung ECs. By contrast, the B cell SFK *BLK* was consistently classified as nonexpressed or leakily expressed in ECs, similar to erythroid, osteoblast, ocular and sex cell genes. These findings are analogous to prior studies reporting that LE cluster transcripts include markers of cell types other than the one under investigation^13,16^. Pseudobulk analysis of scRNA-seq data is increasingly used to account for cell-to-cell heterogeneity in transcription and to overcome challenges in transcript detection at the single-cell level^56^. When pseudobulk analysis or emerging scRNA-seq techniques with higher sensitivity detect unexpected transcripts, implementing methods analogous to those used in the BulkECexplorer may help predict which transcripts arise from leaky versus active transcription.

To evaluate systematically the predictive performance of the GMM and zTPM tools included in the BulkECexplorer, we used a confusion matrix-based approach to score against established EC and non-EC markers. Without notable loss of sensitivity, the GMM- and zTPM-based approaches scored better for specificity than classifying genes based on transcript detection alone (>0 TPM) and slightly better than setting an expression threshold of >1 TPM. The results of this comparison, therefore, provide a rationale for using the 1 TPM threshold to predict which genes are actively transcribed when GMM and zTPM models are unavailable. Nevertheless, the four classification approaches individually have potential weaknesses for interpreting bulk RNA-seq data. Using 0 TPM as a threshold for classification returns many genes with very low transcript levels, most of which likely belong to the leaky transcriptome. GMM classification cannot evaluate all datasets (that is, exclusion of those that are not bimodal), and gene−dataset combinations around the intersection of the LE and HE distributions cannot be confidently classified as ‘active’ or ‘leaky’ (reported as ‘undetermined’ by the BulkECexplorer). zTPM classification depends on preexisting work correlating chromatin accessibility and gene expression data to determine the threshold for active gene expression in a given cell type; here, we have applied the threshold previously obtained for HUVECs to other EC subtypes, but we cannot exclude that it may vary somewhat between subtypes. To overcome the individual limitations of each classification method, the BulkECexplorer returns their results alongside each other, both graphically and numerically (in a summary table). Therefore, viewing results with the ‘transcript >0 TPM’ threshold identifies datasets completely lacking transcripts for a gene of interest from those that contain any level of transcript. In contrast, the GMM and zTPM approaches help predict whether transcript levels are likely biologically relevant. Thus, simultaneously applying multiple classification approaches can help overcome the potential weaknesses of individual approaches for transcript classification.

Transcripts typical of EC-associated cell types were detected across EC subtypes in the BulkECexplorer. Thus, we should consider that ECs freshly isolated from mouse organs for bulk RNA-seq might be contaminated by EC-associated cell types not fully dissociated from ECs during sample preparation. Notwithstanding the technical challenge of preventing such contamination, an alternative explanation arises from a prior study that correlated bulk and scRNA-seq data from lung, brain and heart ECs to conclude that the endothelium genuinely expresses some transcripts characteristic of parenchymal cells^7^. The prevalence of host organ transcripts in ECs can be readily surveyed with the BulkECexplorer. A lower number of non-EC transcripts in HUVEC datasets may reflect that culture methods remove transcripts from non-EC contaminants while also eliminating host organ context. These considerations highlight the importance of interrogating bulk RNA-seq data alongside scRNA-seq and proteomic data to evaluate the extent of host cell transcriptional mimicry while considering the technical challenge of obtaining a pure EC population for bulk RNA-seq.

In summary, the BulkECexplorer interrogates endothelial gene expression data through an online interface that is readily accessible without prior bioinformatics expertise. By predicting which EC genes are expressed at biologically relevant levels, including in EC subtypes commonly used for in vitro research, BulkECexplorer analysis will synergize with scRNA-seq-based analysis to help prioritize genes for functional studies. Such knowledge should be helpful when designing interventional studies in vitro, for example, when candidate genes have been identified through genome-wide association studies or two-hybrid assays. Interrogating the BulkECexplorer may also help affirm the EC expression of genes whose transcripts are not readily detected in scRNA-seq datasets for technical reasons and can be combined with scRNA-seq to investigate unexpected EC transcripts. Beyond the usefulness for the wider vascular community, our resource provides a blueprint for developing analogous tools for other cell types.

## Methods

### Bulk RNA-seq dataset selection

Bulk RNA-seq datasets were retrieved from the ENA in July 2020. To identify relevant datasets, we queried the archive for the following terms: ‘HUVEC’, ‘HDMEC’, ‘HMVEC’ (human microvascular EC), ‘dermal endothelial’, ‘retinal endothelial cells’, ‘brain endothelial cells’ and ‘mouse lung endothelial cells’. Our queries returned 195 RNA-seq projects whose datasets we individually examined to determine their suitability for our analysis. Only datasets generated by bulk or RiboTag RNA-seq were retained for analysis. We included only mouse datasets for brain, retina and lung ECs. A small number of projects that contained datasets with multiple run identifiers were excluded to simplify and streamline the downstream analysis. We also excluded datasets that were erroneously tagged as endothelial but did not include an EC type or were ambiguous in their description. As we wished to examine the ‘basal’ transcriptome of ECs, we excluded datasets from rapidly growing and remodeling embryos. For the same reason, we excluded datasets from cells that had been stimulated (for example, with a small molecule or by hypoxia) and/or had been genetically or functionally modified (for example, by gene deletion, protein overexpression or immortalization). However, we retained datasets in these projects that were derived from control cells (for example, vehicle-stimulated or small interfering RNA control-transfected ECs). A total of 264 datasets with a unique identifier passed this exclusion stage (Supplementary Table 1). After alignment and transcript quantification, we further excluded datasets that did not express >1 TPM of the core endothelial markers *KDR* and *CDH5*. We additionally excluded the project PRJEB14163, which contained datasets with absent or low *KDR* expression and low read number. A total of 240 datasets with a unique identifier from 59 projects passed this exclusion stage and were processed for further analysis (Supplementary Table 1).

### Bulk RNA-seq transcript quantification

FASTQ files were downloaded from the ENA. Reads were aligned to the Genome Reference Consortium Human Build 38 patch release 13 (GRCh38.p13) and Mouse Build 38 patch release 6 (GRCm38.p6), as appropriate, using HISAT2 (version 2.1.0)^57^. Transcript abundance was quantified using StringTie (version 2.1.3)^58^ with the reference annotation file Homo_sapiens.GRCh38.100.gtf or Mus_musculus. GRCm38.100.gtf, as appropriate (Ensemble). Transcript abundance was recorded as TPM. All subsequent RNA-seq analyses were performed in RStudio using R (version 3.6.1).

### Prediction of active versus leaky transcription based on GMMs

For each RNA-seq dataset in the BulkECexplorer, we log2-transformed its TPM values for all protein-coding genes. We then used the R package ‘Mixtools’ (version 1.2.0) and the ‘normalmixEM’ function for expectation maximization to estimate the parameters of the two Gaussian distributions, termed the active and leaky distributions (*μ*_1_,*σ*_1_ and *μ*_2_,*σ*_2_, where *μ* = mean, *σ* =s.d.). For each dataset, the ‘normalmixEM’ function returned a class of ‘mixEM’ data, which included both the parameters of the fitted Gaussian distributions (*μ, σ*) and the posterior probabilities for each gene belonging to each component (that is, probability of a gene belonging to either the active or leaky Gaussian distribution). As this method was applied to each dataset individually, the model was optimized to each dataset’s transcript distribution within the parameters of a two-component GMM. We used the probability of belonging to the higher Gaussian distribution, termed *P*(active), to classify genes as either likely actively expressed (*P*(active) > 0.67) or likely leakily expressed (*P*(active) < 0.33). Genes with a *P*(active) value between these thresholds were assigned as undetermined. Datasets that displayed an apparent unimodal log_2_(TPM) distribution without a left shoulder or required fitting with more than two components/distributions were excluded from this analysis because low- and high-expression gene clusters could not be readily identified (*n* = 42 excluded, *n* = 198 analyzed; Supplementary Table 1). The BulkECexplorer online display provides the resulting information for each EC subtype as the total number of datasets in which a queried gene is classified as leaky, undetermined or active as a stacked bar chart and numerically in the summary table. The percentage of datasets in which the gene was classified as active for that EC subtype is listed below the corresponding bar. The percentage of datasets in which the gene was classified as active or leaky for all EC subtypes combined is shown above the bar graph.

### Prediction of active versus leaky transcription based on zTPM scores

zTPM scores were calculated using the zFPKM function in the R ‘zFPKM’ package^9^. Briefly, this function takes gene expression data in TPM or FPKM form and calculates the distribution of expression values by the kernel density estimate (KDE). The function then builds a half-Gaussian distribution to the right of the KDE peak, assigning *μ* as the KDE maximum. The half-Gaussian is mirrored to create a full Gaussian distribution, and the parameters of this full Gaussian distribution are used to standardize gene expression values to a *z* score termed zFPKM or zTPM, depending on the original unit of gene expression value. Correlation analysis between zTPM and zFPKM was performed with R ‘cor.test’. zFPKM thresholds for selecting genes with biologically relevant levels of expression have been published for a range of cell types, and we used the threshold for HUVECs in our analysis (−2.38 zFPKM)^9^. Datasets that displayed an apparent unimodal log_2_(TPM) distribution without evidence of a left shoulder were excluded from this analysis because the zTPM transformation relies on identifying a high-expression Gaussian distribution (*n* = 20 excluded, *n* = 220 included; Supplementary Table 1). The BulkECexplorer online display provides the resulting information for each EC subtype as the total number of datasets in which a queried gene is classified as expressed above a −2.38 zTPM threshold, both as a stacked bar chart and in numerical form in the summary table. The percentage of datasets in which the gene was classified as expressed above the threshold is listed below the corresponding bar. The percentage of datasets in which the gene was classified as expressed above the threshold across all EC subtypes is shown above the bar graph.

### Evaluation of predictive performance

We evaluated the predictive performance of the BulkECexplorer with a confusion matrix (Supplementary Table 3). As the BulkECexplorer does not provide a single summary classification for a gene of interest across all datasets but instead classifies each gene within each dataset, we considered the individual gene classifications from each dataset (gene−dataset combinations) as predicted values. Thus, BulkEC-explorer data from each of the four classification methods (‘transcript >0 TPM’, ‘transcript >1 TPM’, GMM and zTPM) were used as the input, whereby genes with TPM = 0 were considered predicted negatives. gene−dataset combinations that were classified as ‘undetermined’ by the GMM method were not included in the confusion matrix analysis. The predicted values were tested against a set of actual positive markers (*n* = 37) and a set of actual negative markers (*n* = 109). Actual positive markers were selected as genes with established expression and function within ECs (Supplementary Table 2). Actual negative markers were selected as known markers of non-EC cell types, including neurons, bone cells, germ cells, adipose cells, immune cells, ocular cells, skeletal muscle cells, epithelial cells, pericytes and smooth muscle cells (Supplementary Table 2). Sensitivity was calculated as the number of correctly predicted positives over the total number of gene−dataset combinations for actual positive markers. Specificity was calculated as the number of correctly predicted negatives over the total number of gene−dataset combinations for actual negative markers. These performance metrics were calculated for each classification method separately, both for all EC subtypes together and for the individual EC subtypes. After identifying six presumed actual negative markers in the HUVEC proteome (Extended Data Table 1), the list of actual negative markers was refined for a second run (*n* = 103; actual positive markers retained as *n* = 37). A third run was carried out after the removal of markers of EC-interacting cell types (Extended Data Table 1) from the list of actual negative markers (*n* = 49; actual positive markers retained as *n* = 37). Analyses included all datasets eligible for each classification method (threshold *n* = 240, zTPM *n* = 220, GMM *n* = 198).

### BulkECexplorer app

The R ‘Shiny’ (version 1.7.4.1) and ‘shinydashboard’ (version 0.7.2) packages were used to create the BulkECexplorer Web application (https://ruhrberglab.shinyapps.io/BulkECexplorer).

### scRNA-seq analysis

Raw count data for the EC atlas^33^ were downloaded from the EC atlas Shiny app (https://endotheliomics.shinyapps.io/ec_atlas/). R objects containing count data from Tabula Muris^34^ were downloaded from https://figshare.com/articles/dataset/Robject_files_for_tissues_processed_by_Seurat/5821263. The human dermal EC dataset was downloaded from the BIG Data Center (https://bigd.big.ac.cn/)^35^, and the human trachea dataset was downloaded from the Human Cell Landscape project in the Gene Expression Omnibus of the National Center for Biotechnology Information (https://www.ncbi.nlm.nih.gov/geo/)^36^. Analyses were performed with RStudio (version 1.3.1056) using R (version 4.2.0). The raw gene expression matrices (unique molecular identifier counts per gene per cell) were filtered, normalized and clustered using the R package Seurat (version 3.2.3)^59,60^. Cells containing <200 feature counts were omitted, except for the Tabula Muris data R objects, which had been preprocessed to exclude cells with <500 feature counts. Genes detected in fewer than three cells were removed. Downstream analysis included data normalization (‘LogNormalize’ method and scale factor of 10,000) and variable gene detection (‘vst’ selection method, returning 2,000 features per dataset). For each organ, ECs were identified as described^38^. Principal component (PC) analysis was performed on variable genes, and the optimal number of PCs for each dataset was chosen using the elbow plot. The selected PCs were used for Louvain graph-based clustering at a resolution of 0.3. Uniform Manifold Approximation and Projection (UMAP) was chosen as a nonlinear dimensionality reduction method, and each relevant gene was then examined using the ‘FeaturePlot’ and ‘VlnPlot’ functions. Cluster cell identity was assigned by manual annotation based on known marker genes, followed by a subset selection of clusters containing *PECAM1*-positive ECs.

## Extended Data

**Extended Data Table 1 |:** Detection of selected EC and non-EC markers in the HUVEC proteome and BulkECexplorer transcriptomes.

	Gene symbol	Expected cell type^1^	Protein info^1^	HUVEC proteome^2^	BulkECexplorer-constituent EC subtypes															Comments and potential explanations for unexpected findings
					HUVEC	HDMEC	Lung EC	Brain EC	Retina EC	HUVEC	HDMEC	Lung EC	Brain EC	Retina EC	HUVEC	HDMEC	Lung EC	Brain EC	Retina EC	
				Protein levels (Log2 iBAQ)	Median in TPM					GMM classification (% datasets with active expression in eligible datasets)					zTPM analysis (% datasets with active expression in eligible datasets)					
**EC genes**	*KDR*	ECs (pan)	transmembrane receptor	21.15	108.9	104.6	80.1	288.0	280.9	100	100	100	100	100	100	100	100	100	100	used as selection criterion for dataset inclusion
	*CDH5*	ECs (pan)	junctional protein	24.30	822.1	204.7	728.1	191.8	240.8	100	100	100	100	100	100	100	100	100	100	used as selection criterion for dataset inclusion
	*PECAM1*	ECs (pan)	junctional protein	25.32	573.7	715.3	501.4	708.2	281.0	100	100	100	100	100	100	100	100	100	100	positive control for BulkECexplorer analysis
	*TEK*	ECs (pan)	transmembrane receptor	19.87	67.1	61.9	99.8	278.4	215.8	100	100	100	100	100	100	100	100	100	100	
	*ERG*	ECs (pan)	transcription factor	22.39	134.2	100.4	111.4	75.3	76.3	100	100	100	100	100	100	100	100	100	100	
	*PDGFB*	ECs (pan)	secreted factor	20.69	97.1	77.4	138.6	154.2	228.2	100	100	100	100	100	100	100	100	100	100	
	*VWF*	ECs (pan)	secreted factor	24.91	926.6	541.8	52.7	103.1	206.9	100	100	92	98	100	100	100	93	98	100	
	*CLDN5*	ECs (pan)	junctional protein	ND	101.3	488.8	486.5	3755.1	889.9	98	100	100	100	100	100	100	100	100	100	
	*TAL1*	ECs (pan)	transcription factor	ND	30.0	15.2	11.7	7.0	12.4	100	100	100	98	86	100	100	100	98	100	
	*KLF4*	ECs	transcription factor	ND	2.6	4.2	41.2	215.1	42.9	53	71	100	100	100	84	86	100	100	100	sheer stress regulator, low levels in static EC cultures
	*PLVAP*	ECs	fenestrae and caveolae	ND	13.6	66.9	231.4	15.9	8.3	90	86	100	95	86	98	100	100	93	72	fenestrated EC marker, low levels in blood brain and retina barrier ECs
	*MFSD2A*	ECs (brain, retina)	transmembrane transporter	ND	2.7	2.3	1.2	401.4	151.9	65	64	25	95	100	84	93	47	96	100	blood brain and retina barrier EC marker
**non-EC genes**	*LENEP*	ocular	lens protein	ND	0.1	0.2	0.2	0.6	0.8	0	0	0	18	0	0	7	7	39	11	
	*BGLAP*	osteoblast	secreted factor	ND	0.1	0.4	0.5	0.3	0.6	2	14	0	0	0	6	43	0	7	0	
	*SPACA4*	sex cell	transmembrane receptor	ND	0.1	0.1	0.4	0.2	0.7	0	0	0	0	0	0	14	7	2	0	
	*MYOG*	skeletal muscle	transcription factor	ND	0.1	0.0	0.0	0.4	0.2	0	0	0	0	0	0	0	0	2	0	
	*CDH1*	Epithelial cells	junctional protein	ND	0.0	4.4	24.6	1.4	0.3	0	43	8	35	0	0	43	20	39	6	
	*KLF1*	Erythroid cells	transcription factor	ND	0.1	0.1	0.4	0.3	0.9	0	0	0	0	0	0	0	0	2	0	used as negative control for BulkECexplorer analysis
**non-EC genes (contamination)**	*ITGAM*	Myeloid cells	transmembrane receptor	ND	0.1	0.1	0.7	1.2	68.1	1	0	17	30	100	2	0	40	37	78	marker of EC interacting cell type
	*GFAP*	Astrocytes	cytoskeleton	ND	0.1	0.1	0.1	12.4	4.7	1	0	0	73	14	1	0	0	83	89	marker of EC interacting cell type
	*OPALIN*	Olygodendr ocytes	transmembrane protein	ND	0.0	0.0	0.0	3.6	0.0	0	0	0	45	0	0	0	0	46	0	marker of EC interacting cell type
	*SOX2*	Neural cells	transcription factor	ND	0.1	0.0	0.8	5.9	6.7	3	0	8	73	43	7	7	7	78	100	marker of EC interacting cell type
	*DES*	PCs/SMCs	cytoskeleton	ND	0.1	0.0	11.5	7.0	0.8	0	0	50	85	0	0	0	53	91	17	marker of EC interacting cell type
**non-EC genes (EC expression)**	*PNPLA2*	adipose tissue	enzyme	16.62	59.6	64.5	26.7	80.3	38.3	100	100	100	100	100	100	100	100	98	100	non-EC marker expressed in all BulkECexplorer EC subtypes
	*TAGLN*	PCs/SMCs	cytoskeleton	26.43	10.1	31.6	74.3	21.9	4.0	98	93	75	93	43	100	100	80	93	78	non-EC marker expressed in most BulkECexplorer EC subtypes
	*MAP2*	Neural cells	cytoskeleton	20.33	10.7	2.3	0.3	4.2	219.8	96	79	40	75	100	100	100	47	91	100	non-EC marker expressed in most BulkECexplorer EC subtypes
	*GAD1*	Neural cells	enzyme	14.51	0.1	0.1	0.1	3.3	48.7	0	0	0	63	100	0	0	7	80	94	non-EC marker expressed in some BulkECexplorer EC subtypes
	*GLUL*	Neural cells	enzyme	18.51	51.4	53.8	92.2	899.8	899.3	100	100	100	100	100	100	100	100	100	100	non-EC marker expressed in all BulkECexplorer EC subtypes
	*GAPDHS*	Neural cells	enzyme	25.85	0.1	0.3	0.7	0.7	5.6	0	0	14	0	17	0	0	20	11	67	not in BulkECexplorer: proteome misidentified?
	*NES*	Neural cells	cytoskeleton	26.35	142.5	68.3	68.0	119.4	44.7	100	100	100	100	100	100	100	100	100	100	non-EC marker expressed in all BulkECexplorer EC subtypes

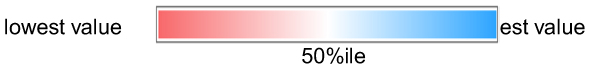

1 https://www.proteinatlas.org/; https://www.uniprot.org/

2 PMID: 30983154

Protein and transcript levels for the indicated genes, as detected in a published HUVEC proteome and in BulkECexplorer, respectively, together with their GMM- and zTPM-based classification. The table broadly groups genes as follows: expressed in EC (EC genes) and expressed in cells other than ECs (non-EC genes), including markers of non-EC-genes that interact with ECs (non-EC genes, EC interacting cells). Note markers for presumed non-ECs for which EC expression was identified here (presumed non-EC genes, EC expression). Information on the cellular profile of gene expression and gene product function were confirmed by surveying the Human Protein Atlas (https://www.proteinatlas.org/) and UniProt (https://www.uniprot.org/).

## Supplementary Material

Supplemental data tables.xls

Supplementary Material

## Figures and Tables

**Table 1:** Prevalence of SFK expression in ECs from scRNA-seq data

Dataset name	Species	Organ	Method	EC selection	No. of cells	% ECs with detectable transcript levels									
						*Pecam1*	*Yes1*	*Src*	*Fyn*	*Lyn*	*Fgr*	*Hck*	*Lck*	*Blk*	*Klf1*
EC atlas	Mm	Brain	Droplet (10x)	FACS	3,724	79.08	11.73	1.45	13.53	14.34	ND	ND	ND	ND	ND
EC atlas	Mm	Lung	Droplet (10x)	FACS	5,152	84.96	12.09	ND	15.47	30.20	ND	ND	ND	ND	ND
EC atlas	Mm	Heart	Droplet (10x)	FACS	4,525	72.04	15.47	ND	20.57	20.66	ND	ND	ND	ND	ND
						*Pecam1*	*Yes1*	*Src*	*Fyn*	*Lyn*	*Fgr*	*Hck*	*Lck*	*Blk*	*Klf1*
Tabula Muris	Mm	Brain	Smart-seq2	Seurat	733	90.45	22.37	5.59	32.06	33.70	0.41	3.14	3.00	1.36	0.27
Tabula Muris	Mm	Lung	Smart-seq2	Seurat	698	96.13	16.91	1.15	20.34	35.24	0.86	2.58	5.16	0.14	0.14
Tabula Muris	Mm	Heart	Smart-seq2	Seurat	1,376	89.03	22.02	0.51	34.01	44.91	0.58	3.85	5.31	1.02	0.15
Tabula Muris	Mm	Trachea	Smart-seq2	Seurat	112	100.00	50.00	1.79	41.96	47.32	1.79	0.89	3.57	3.57	0.89
						*PECAM1*	*YES1*	*SRC*	*FYN*	*LYN*	*FGR*	*HCK*	*LCK*	*BLK*	*KLF1*
Not applicable	Hs	Dermis	Droplet (10x)	FACS	47,668	79.56	25.48	0.14	20.32	14.75	5.63	0.14	0.02	0.02	ND
						*PECAM1*	*YES1*	*SRC*	*FYN*	*LYN*	*FGR*	*HCK*	*LCK*	*BLK*	*KLF1*
Human Cell Landscape	Hs	Trachea	Microwell	Seurat	2,029	72.70	75.21	11.34	5.47	3.55	2.27	0.15	0.05	0.05	ND

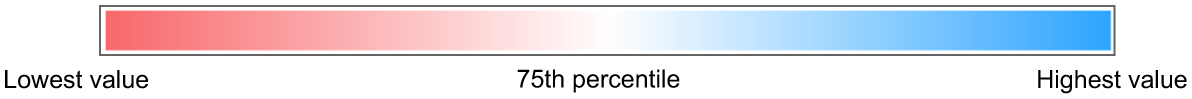

The table shows the percentage of ECs with detectable transcript levels for SFKs relative to the core EC marker *PECAM1* and the non-EC, erythrocyte marker *KLF1* in mouse and human ECs from the indicated organs in the indicated scRNA-seq datasets. EC selection was achieved either by FACS or through clustering with Seurat. Mm, *Mus musculus*; Hs, *Homo sapiens*; ND, not detected.

**Table 2:** Average transcript levels for SFKs in ECs from scRNA-seq compendia

Dataset name	Species	Organ	Method	EC selection	No. of cells	Average normalised gene expression									
						*Pecam1*	*Yes1*	*Src*	*Fyn*	*Lyn*	*Fgr*	*Hck*	*Lck*	*Blk*	*Klf1*
EC atlas	Mm	Brain	Droplet (10x)	FACS	3,724	6.7179	0.4745	0.0474	0.4938	0.5524	ND	ND	ND	ND	ND
EC atlas	Mm	Lung	Droplet (10x)	FACS	5,152	11.3680	0.5605	ND	0.6237	1.4795	ND	ND	ND	ND	ND
EC atlas	Mm	Heart	Droplet (10x)	FACS	4,525	8.4348	0.8162	ND	1.1266	1.1512	ND	ND	ND	ND	ND
						*Pecam1*	*Yes1*	*Src*	*Fyn*	*Lyn*	*Fgr*	*Hck*	*Lck*	*Blk*	*Klf1*
Tabula Muris	Mm	Brain	Smart-seq2	Seurat	733	0.8609	0.1577	0.0813	0.6224	0.6411	0.0055	0.0142	0.0019	0.0003	0.0011
Tabula Muris	Mm	Lung	Smart-seq2	Seurat	698	4.3473	0.1959	0.0288	0.4622	1.2627	0.0005	0.0013	0.0141	0.0000	0.0128
Tabula Muris	Mm	Heart	Smart-seq2	Seurat	1,376	11.3991	0.4123	0.0028	1.2679	1.8057	0.0001	0.0015	0.0138	0.0002	0.0001
Tabula Muris	Mm	Trachea	Smart-seq2	Seurat	112	14.7314	0.7976	0.0048	0.8905	1.3022	0.0285	0.0001	0.0070	0.0009	0.0002
						*PECAM1*	*YES1*	*SRC*	*FYN*	*LYN*	*FGR*	*HCK*	*LCK*	*BLK*	*KLF1*
Not applicable	Hs	Skin	Droplet (10x)	FACS	47,668	8.0312	0.8740	0.0041	0.6546	0.4780	0.1588	0.0040	0.0006	0.0006	ND
						*PECAM1*	*YES1*	*SRC*	*FYN*	*LYY*	*FGR*	*HCK*	*LCK*	*BLK*	*KLF1*
Human Cell Landscape	Hs	Trachea	Microwell	Seurat	2,029	9.8724	3.6929	0.0895	0.6189	0.3905	0.2931	0.0111	0.0030	0.0090	ND

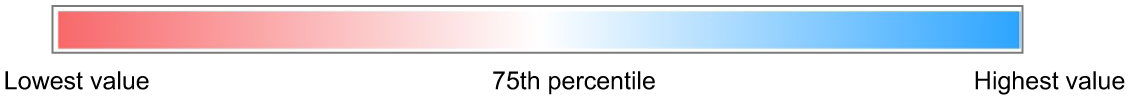

The table shows the average transcript Levels for SFKs relative to the core EC marker *PECAM1* and the non-EC, erythrocyte marker *KLF1* across all cells in the EC cluster in mouse and human ECs from the indicated organs. EC selection was achieved either by FACS or through clustering with Seurat. Normalization of gene expression was performed with Seurat.

**Table 3:** Predictive performance of the BulkECexplorer

Subtype and classification method		Original list (146 markers)		Refined list (140 markers)— removal of actual negatives present in the HUVEC proteome		Revised list (86 markers)— removal of markers for host cells interacting with ECs	
EC subtype	Classifier	Sensitivity	Specificity	Sensitivity	Specificity	Sensitivity	Specificity
All	>0 TPM	1.00	0.56	1.00	0.58	1.00	0.70
	>1 TPM	0.98	0.79	0.98	0.82	0.98	0.90
	GMM	0.99	0.84	0.99	0.87	0.99	0.94
	zTPM	0.99	0.79	0.99	0.82	0.99	0.90
HUVEC	>0 TPM	1.00	0.62	1.00	0.65	1.00	0.71
	>1 TPM	0.98	0.89	0.98	0.92	0.98	0.94
	GMM	0.99	0.90	0.99	0.93	0.99	0.95
	zTPM	0.99	0.88	0.99	0.91	0.99	0.93
HDMEC	>0 TPM	1.00	0.62	1.00	0.64	1.00	0.71
	>1 TPM	0.97	0.86	0.97	0.89	0.97	0.92
	GMM	0.98	0.86	0.98	0.89	0.98	0.92
	zTPM	0.98	0.80	0.98	0.83	0.98	0.87
Mouse lung EC	>0 TPM	1.00	0.62	1.00	0.64	1.00	0.75
	>1 TPM	0.98	0.80	0.98	0.82	0.98	0.88
	GMM	0.97	0.85	0.97	0.87	0.97	0.93
	zTPM	0.98	0.80	0.98	0.82	0.98	0.88
Mouse brain EC	>0 TPM	0.99	0.44	0.99	0.46	0.99	0.68
	>1 TPM	0.99	0.64	0.99	0.66	0.99	0.85
	GMM	0.99	0.67	0.99	0.70	0.99	0.90
	zTPM	0.99	0.62	0.99	0.64	0.99	0.83
Mouse retina EC	>0 TPM	1.00	0.37	1.00	0.39	1.00	0.60
	>1 TPM	0.99	0.48	0.99	0.51	0.99	0.75
	GMM	1.00	0.62	1.00	0.65	1.00	0.90
	zTPM	0.97	0.58	0.97	0.61	0.97	0.85

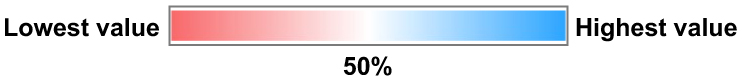

The table shows specificity and sensitivity metrics calculated for known EC-functional genes (actual positives) and known non-EC genes (actual negatives) with the GMM or zTPM classification models and thresholds of >0 TPM or >1 TPM. Each eligible dataset for each of the four classification approaches was treated as an individual test (*n* = 240 for transcript level >0 TPM or >1 TPM, *n* = 220 for zTPM, *n* = 198 for GMM). Original run: *n* = 37 actual positives and *n* = 109 actual negatives; refined run after subtracting six HUVEC proteome-expressed genes from the list of actual negatives: *n* = 37 actual positives and *n* = 103 actual negatives; amended run after subtracting markers of neural, glial, mural and myeloid cells from the list of actual negatives: *n* = 37 actual positives and *n* = 49 actual negatives.

## Data Availability

Publicly available datasets were analyzed in this study: adult mouse scRNA-seq (https://tabula-muris.ds.czbiohub.org/;GSE109774), adult mouse EC scRNA-seq (EC atlas; https://endotheliomics.shinyapps.io/ec_atlas/;E-MTAB-8077), human dermal EC scRNA-seq (https://bigd.big.ac.cn/; PRJCA002692), human trachea EC scRNA-seq (Human Cell Landscape; https://db.cngb.org/search/?q=CNP0000325;GSE134355). For bulk RNA-seq datasets retrieved from the ENA, dataset identifiers can be found in Supplementary Table 1. Data supporting the findings in this study are included in the main article or associated files (Supplementary Tables 2 and 3 and source data for Figs. 2−5). Source data are provided with this paper.

## Reporting summary

Further information on research design is available in the Nature Portfolio Reporting Summary linked to this article.

## Code availability

Code is available at https://github.com/ruhrberg.
