# Supplementary material for: The BulkECexplorer compiles endothelial bulk transcriptomes to predict functional versus leaky transcription
Source: Nat Cardiovasc Res. Author manuscript; Available in PMC 2024 May 3. (PMC7615926; doi:10.1038/s44161-024-00436-w)
Supplement: Supplementary Material [file EMS195816-supplement-Supplementary_Material.pdf]

nature portfolio

Corresponding author(s): Alessandro Fantin and Christiana Ruhrberg

Last updated by author(s): Jan 9, 2024

Reporting Summary

Nature Portfolio wishes to improve the reproducibility of the work that we publish. This form provides structure for consistency and transparency in reporting. For further information on Nature Portfolio policies, see our Editorial Policies and the Editorial Policy Checklist.

Statistics

For all statistical analyses, confirm that the following items are present in the figure legend, table legend, main text, or Methods section.

n/a	Confirmed	
☐	☒	The exact sample size (*n*) for each experimental group/condition, given as a discrete number and unit of measurement
☒	☐	A statement on whether measurements were taken from distinct samples or whether the same sample was measured repeatedly
☐	☒	The statistical test(s) used AND whether they are one- or two-sided<br>*Only common tests should be described solely by name; describe more complex techniques in the Methods section.*
☒	☐	A description of all covariates tested
☒	☐	A description of any assumptions or corrections, such as tests of normality and adjustment for multiple comparisons
☐	☒	A full description of the statistical parameters including central tendency (e.g. means) or other basic estimates (e.g. regression coefficient) AND variation (e.g. standard deviation) or associated estimates of uncertainty (e.g. confidence intervals)
☐	☒	For null hypothesis testing, the test statistic (e.g. *F*, *t*, *r*) with confidence intervals, effect sizes, degrees of freedom and *P* value noted<br>*Give P values as exact values whenever suitable.*
☒	☐	For Bayesian analysis, information on the choice of priors and Markov chain Monte Carlo settings
☒	☐	For hierarchical and complex designs, identification of the appropriate level for tests and full reporting of outcomes
☐	☒	Estimates of effect sizes (e.g. Cohen's *d*, Pearson's *r*), indicating how they were calculated

Our web collection on statistics for biologists contains articles on many of the points above.

Software and code

Policy information about availability of computer code

Data collection	No software was used to collect the data
Data analysis	Analyses were performed with RStudio version 1.3.1056 and 3.6.1, R version 4.2.0, the R packages Seurat v.3.2.0, HISAT2 version 2.1.0, Stringtie version 2.1.3, 'Mixtools' v 1.2.0, 'zFPKM' version 1.24.0, 'shiny' version 1.7.4.1 and 'shinydashboard' version 0.7.2. Code is available at github.com/ruhrberg.

For manuscripts utilizing custom algorithms or software that are central to the research but not yet described in published literature, software must be made available to editors and reviewers. We strongly encourage code deposition in a community repository (e.g. GitHub). See the Nature Portfolio guidelines for submitting code & software for further information.

Data

Policy information about availability of data

All manuscripts must include a data availability statement. This statement should provide the following information, where applicable:

- Accession codes, unique identifiers, or web links for publicly available datasets
- A description of any restrictions on data availability
- For clinical datasets or third party data, please ensure that the statement adheres to our policy

Publicly available datasets were analysed in this study: adult mouse scRNA-seq (https://tabula-muris.ds.czbiohub.org/, GSE109774), adult mouse EC scRNA-seq (EC Atlas; https://endotheliomics.shinyapps.io/ec_at; E-MTAB-8077), human dermal EC scRNA-seq (https://bigd.big.ac.cn/; PRJCA002692), human trachea EC scRNA-seq

(Human Cell Landscape; https://db.cngb.org/search/?q=CNP0000325; GSE134355). For Bulk RNA-seq datasets retrieved from the European Nucleotide Archive (ENA), dataset identifiers can be found in Supplemental Table 1. Data supporting the findings in this study are included in the main article or associated files.

Research involving human participants, their data, or biological material

Policy information about studies with human participants or human data. See also policy information about sex, gender (identity/presentation), and sexual orientation and race, ethnicity and racism.

Reporting on sex and gender	not applicable
Reporting on race, ethnicity, or other socially relevant groupings	not applicable
Population characteristics	not applicable
Recruitment	not applicable
Ethics oversight	not applicable

Note that full information on the approval of the study protocol must also be provided in the manuscript.

Field-specific reporting

Please select the one below that is the best fit for your research. If you are not sure, read the appropriate sections before making your selection.

☒ Life sciences ☐ Behavioural & social sciences ☐ Ecological, evolutionary & environmental sciences

For a reference copy of the document with all sections, see nature.com/documents/nr-reporting-summary-flat.pdf

Life sciences study design

All studies must disclose on these points even when the disclosure is negative.

Sample size	Sample size was not pre-determined. Samples were obtained by quering the European Nucleotide Archive (ENA) in July 2020 for all the RNA-seq datasets including the following terms in their description: 'HUVEC', 'HDMEC', 'HMVEC', 'dermal endothelial', 'retinal endothelial cells', 'brain endothelial cells' or 'mouse lung endothelial cells'
Data exclusions	From the intial selection of 195 RNA-seq projects, single datasets were individually examined to determine their suitability for our analysis. Only datasets generated by bulk or Ribo-tag RNA-seq were retained for analysis. We only included murine datasets for brain, retina and lung ECs. A small number of projects that contained samples with multiple run IDs were excluded to simplify and streamline downstream analysis. We also excluded datasets that were erroneously tagged as endothelial but did not include an EC type or which were ambiguous in their description. As we wished to examine the 'basal' transcriptome of ECs, we excluded samples from rapidly growing and remodelling embryos. For the same reason, we excluded samples that had been stimulated (e.g., with a small molecule or by hypoxia), had been genetically or functionally modified (e.g., by gene deletion, protein overexpression or immortalisation). After alignment and transcript quantification, we further excluded samples that did not express >1 TPM of the core endothelial markers KDR or CDH5. We additionally excluded the project PRJEB14163, which contained samples with absent or low KDR expression and low read number.
Replication	The bioinformatic analyses included this manuscript can be replicated thanks to code availability at github.com/ruhrberg.
Randomization	This is not relevant to our study as we did not divide sample in different experimental groups.
Blinding	This is not relevant to our study as we did not divide sample in different experimental groups.

Reporting for specific materials, systems and methods

We require information from authors about some types of materials, experimental systems and methods used in many studies. Here, indicate whether each material, system or method listed is relevant to your study. If you are not sure if a list item applies to your research, read the appropriate section before selecting a response.

Materials & experimental systems

n/a	Involved in the study	
☒	☐	Antibodies
☒	☐	Eukaryotic cell lines
☒	☐	Palaeontology and archaeology
☒	☐	Animals and other organisms
☒	☐	Clinical data
☒	☐	Dual use research of concern
☒	☐	Plants

Methods

n/a	Involved in the study	
☒	☐	ChIP-seq
☒	☐	Flow cytometry
☒	☐	MRI-based neuroimaging
